# Institutional Anthropomorphic Error

**DOI:** 10.1007/s13347-026-01191-5

**Published:** 2026-09-24

**Authors:** Giles Howdle

**Affiliations:** Department of Philosophy and Religious Studies, Utrecht University, Utrecht, Netherlands

**Keywords:** Anthropomorphism, Artificial Intelligence, AI, Social AI, Institutional Anthropomorphism, Metaphysical Anthropomorphism, Pragmatic Anthropomorphism

## Abstract

Sakr proposes institutional anthropomorphic error (the projection of role-based authority onto AI systems that cannot bear a role’s constitutive obligations) as a third kind of error to be added to my distinction between metaphysical and pragmatic anthropomorphic error. I argue that the proposal is better understood as belonging to a second *category* of anthropomorphism than a third kind of error within an existing one. To ‘anthropomorphise’ a system may mean either seeing it as human-like (anthropomorphic cognition) or presenting it as human-like (anthropomorphic presentation). Metaphysical and pragmatic error are two kinds of error in anthropomorphic cognition; institutional anthropomorphic error, as Sakr defines it, is an error of presentation. A cognitive counterpart can be formulated, but it resolves into a distinctive form of either metaphysical or pragmatic error. Sakr’s contribution is valuable, but it does not require revision of the two-part account.

## Introduction

I am grateful to Sakr (2026) for his article, which builds on the two-way distinction I have drawn between metaphysical and pragmatic anthropomorphic error with respect to social AI systems (SAIs) (Howdle, 2026). Sakr identifies a third form of anthropomorphic error:

Institutional Anthropomorphic Error: positioning or representing an AI system as occupying a human social role in a manner that projects role-based authority onto the system and invites role-based trust in it, although the system cannot bear the role’s constitutive obligations and the arrangement obscures an accountable human or institutional bearer. (2026, p. 3)

Institutional anthropomorphic error is a useful philosophical concept that furthers our understanding of a rapidly emerging and significant concern. However, as Sakr acknowledges, it differs in important respects from the two errors I have described. My aim here is to clarify the significance of those differences.

I claim (1) that we need to distinguish two senses of anthropomorphism and two related *categories* of anthropomorphic error; (2) that the two *kinds* of error I have identified fall under the same *category*, while institutional anthropomorphic error as Sakr describes it falls under a different *category*; and (3) that we can reconceive institutional anthropomorphic error so that it belongs in the same category as metaphysical and pragmatic anthropomorphic error, but that doing so does not require revision of the two-part account I have defended.

## Two Senses of Anthropomorphism

There is a sense in which a person ‘anthropomorphises’ a system by *seeing* it as human-like. There is another in which a person ‘anthropomorphises’ a system by *presenting* it as human-like. Call this the difference between *anthropomorphic cognition* and *anthropomorphic presentation*. Both can be mistakes, but they are two different categories of mistake.

Institutional anthropomorphic error, as Sakr defines it, is a mistake with respect to the ‘positioning or representing’ of an AI system. It is an error of anthropomorphic presentation.

The distinction I have drawn between metaphysical and pragmatic anthropomorphic error, by contrast, is a distinction between two kinds of error in anthropomorphic cognition. Metaphysical error is a matter of being committed to ontological falsehoods with respect to an SAI (e.g. non-existent machine mental states), while pragmatic error is a matter of adopting the intentional stance toward an SAI when another interpretive strategy would be better. These are bad ways of seeing and thinking about SAIs, not bad ways of presenting them. Both pertain to erroneous anthropomorphic cognition.

My paper did briefly draw some normative conclusions about anthropomorphic presentation, but without theorising it. Sakr does a great deal to develop and clarify these normative concerns, and his emphasis on the social roles and offices SAIs occupy, or are taken to occupy, and on their normative significance, is a substantial contribution. Introducing a system as ‘your clinical assistant’ is not equivalent to saying that the model believes p; it places the system in a recognised social role rather than attributing a state to it.

I do not, however, agree with Sakr’s way of framing his contribution. He proposes institutional anthropomorphic error as a *third form* of error, to be added to my two-part taxonomy. But my taxonomy concerns errors of anthropomorphic cognition, whereas his concerns an error of anthropomorphic presentation (Table 1).

**Table 1:** Two categories of anthropomorphism and their errors

Category of Anthropomorphism	Kind of Error	Description
Anthropomorphic Cognition	Metaphysical (Howdle, 2026)	Commitment to ontological falsehoods.
	Pragmatic (Howdle, 2026)	Intentional stance adopted where alternative strategy would better serve one’s interpretive aims.
Anthropomorphic Presentation	Institutional (Sakr, 2026)	Role-based authority projected onto a system that lacks the constitutive competences to bear that authority.

## A Cognitive Institutional Anthropomorphic Error?

I now wish to consider if there is a *cognitive* version of Sakr’s institutional anthropomorphic error, and, if so, whether it should be adopted as a third kind of cognitive anthropomorphic error.

Here is a proposal:

Cognitive Institutional Anthropomorphic Error: thinking of an AI system as occupying a human social role that holds role-based authority and role-based trust, although the system cannot bear the role’s constitutive obligations and the arrangement obscures an accountable human or institutional bearer.

I suspect this kind of cognitive error occurs regularly and that it matters for many of the reasons Sakr suggests. However, I also suspect my two-part taxonomy can accommodate it without modification. To see why, consider what might be happening when someone engages in cognitive institutional anthropomorphic error.

A *metaphysical* institutional anthropomorphic error would be to commit oneself to an ontological falsehood. Specifically, one’s thought or talk would commit one to the existence of an SAI that is occupying a social role or office it does not (perhaps cannot), in fact, occupy. Or, stated in terms of presupposition error, a metaphysical institutional error would consist in thinking about the system in ways that *presuppose* normative competences and powers the SAI lacks.

A *pragmatic* institutional anthropomorphic error would consist in selecting a suboptimal interpretive stance. Consider a user who treats a chatbot *as if* it occupied the office of a therapist, without committing herself to the claim that it does. If the interaction proves harmful, she directs her complaint at the chatbot (as one might to a therapist) rather than at whoever authorised and reviewed its deployment. Her aim of recourse for bad therapeutic advice would have been better served by some alternative interpretive stance.

## Conclusion

Sakr’s concept of institutional anthropomorphic error is a genuine contribution. But it does not, I think, demonstrate that the two-way distinction between metaphysical and pragmatic anthropomorphism requires an institutional sibling. Understood as an error of *presentation*, institutional anthropomorphic error pertains to a different *category* of anthropomorphism, and a different category of anthropomorphic error. Understood instead as an error of *cognition*, I suspect it reduces to a distinctive form of metaphysical or pragmatic anthropomorphic error.

## Data Availability

Not applicable.
